# Knowledge, experience, and perception of molar incisor hypomineralisation among dentists in the metropolitan area of Mexico City: a cross-sectional study

**DOI:** 10.1186/s12903-023-03754-w

**Published:** 2023-12-19

**Authors:** José Francisco Gómez-Clavel, Fernando Yair Sánchez-Cruz, Ximena Paola Santillán-Carlos, Martha Patricia Nieto-Sánchez, Ximena Vidal-Gutiérrez, Álvaro Edgar González-Aragón Pineda

**Affiliations:** 1 Laboratory of Research in Education and Dentistry, Faculty of Higher Studies Iztacala (FES), National Autonomous University of Mexico, (UNAM), Mexico City, Mexico; 2 Specialization in Pediatric Stomatology, Faculty of Higher Studies Iztacala (FES), National Autonomous University of Mexico, (UNAM), Mexico City, Mexico; 3 Faculty of Health Sciences, Autonomous University of Baja California, Mexicali, Mexico; 4 Laboratory of Public Health Research, Iztacala, Faculty of Higher Studies (FES), National Autonomous University of Mexico (UNAM), Mexico City, Mexico; 5 Laboratorio de Investigación en Educación y Odontología, Facultad de Estudios Superiores Iztacala, Universidad Nacional Autónoma de México, Avenida de los Barrios 1, Los Reyes Iztacala, Tlalnepantla, 54090 México

**Keywords:** Molar-incisor hypomineralisation, Knowledge, Perception, General dental practitioner, Paediatric dentist, México, Cross-sectional study

## Abstract

**Background:**

Molar-incisor hypomineralisation (MIH) is the most common developmental abnormality observed in teeth. Being a relatively new condition, its treatment can present a challenge for the dentist. There is currently no study available that has evaluated the knowledge of Mexican dental personnel. This study aimed to evaluate the knowledge, experience, and perceptions of dental surgeons regarding the detection, assessment, and treatment of MIH in the metropolitan area of Mexico City.

**Methods:**

A cross-sectional study was designed. Dentists from Mexico City and its metropolitan area were invited through social networks to answer a questionnaire of 30 questions related to MIH. Participants were classified into general practice dentists, paediatric dentists, and other speciality dentists. Pearson’s chi-square test was used for data analysis.

**Results:**

The questionnaire was answered by 391 dentists. A total of 86% (338 out of 391) of them identified MIH lesions, while 84% of them reported having observed MIH lesions in their practice. The most frequently observed lesions were yellow-brown opacities which accounted for 47% of the lesions, 46% were white opacities, while only 7% were observed as post-eruptive fractures in the enamel as part of the manifestations of MIH. The most frequently reported problem in the management of teeth with MIH was insufficient training for treating children with MIH. A total of 84% of dentists stated that they would like more information on the treatment of MIH lesions.

**Conclusions:**

Most of the surveyed dentists recognised MIH and reported having observed MIH lesions in their practice. Most of the dentists indicated that the main problem for the management of the MIH is the lack of training.

## Background

Molar-incisor hypomineralisation (MIH) is an enamel disorder characterised by the presence of delimited opaque areas of systemic origin that affect one or more first permanent molars with or without involvement of the incisors [1]. These lesions are called qualitative defects owing to their lower mineral content; however, the lack or decrease in the enamel structure cannot be clinically observed. They can detach in the areas of pressure due to occlusal forces, causing the formation of areas of exposed dentin along with those of plaque retention, thereby leading to caries development. Therefore, early diagnosis is important [1].

Clinically, MIH lesions are characterised as delimited opaque areas in the enamel of the first permanent molars, incisors, and second deciduous molars. The colour of the lesions can range from white to yellow-brown. They present usually asymmetrically and with differing severity in the same subject [2].

The reported prevalence of MIH ranges from 2.4 to 40.2% [3], with a mean global prevalence of 12.9% [4], to 14.2% [5]. In Mexico, the reported prevalence varies from 12.4 to 42.4%, with a mean of 28.1% [6–12].

The aetiology of MIH is uncertain; recently, a genetic factor has been associated with it [13]. Several environmental agents or medical conditions during pregnancy and lactation, as well as a recently discovered genetic factor, have been documented. These agents or conditions disrupt the function of ameloblasts during the maturation stage of amelogenesis [14].

The treatment of teeth with MIH lesions can be challenging for the dentist, although successful preventive and treatment options have been proposed and established [15]. In anterior teeth, the problem is mainly cosmetic, whereas, in molars, extensive caries may develop due to the breakdown of hypomineralised enamel [16]. Therefore, the early identification of the teeth affected by MIH is key for the treatment of the affected molars since children avoid oral hygiene procedures owing to the presence of hypersensitivity. The choice of appropriate treatment depends on the severity of the defects and the patient’s age. The European Academy of Paediatric Dentistry recommends the use of all available treatment options, although in severe cases scheduled extractions should be considered [17].

As detection, assessment, and treatment of MIH constitute an emerging oral health problem, questionnaires that aim to assess the dentists’ knowledge, experience, and perception of the disease have been circulated globally [18–30]. The results of these surveys allow us to assess the dentists’ perception of the prevalence, aetiology, and experience with this type of lesion, thereby enabling them to use different approaches for patient care at both, the public and private health care level, and pay attention to the necessary curricular changes and meet the training needs of professionals. However, there is a lack of adequate information on MIH in Mexico. Therefore, the objective of this study is to explore the knowledge, experience, and perception of dental surgeons about the detection, assessment, and treatment of MIH in the metropolitan area of Mexico City.

## Methods

A cross-sectional study was designed, and an online questionnaire developed on the Google platform (Google Forms Questionnaire) was used. The study was approved by the Ethics Commission of the Faculty of Higher Studies, Iztacala, National Autonomous University of Mexico (CE/FESI/112,019/1344. 11/25/2019). All participants gave their informed consent for disseminating the results of the survey.

### Sample

The sample population consisted of dentists whose offices were in Mexico City and its metropolitan area, teachers assigned to schools, and groups of dentists linked to social networking groups. Only dentists with postal codes corresponding to the area of interest were included in the study.

A total of 1032 invitations for participation in the study were sent to dentists residing in Mexico City and the metropolitan area, between October 18, 2021, to January 27, 2022. The questionnaire was disseminated (Google Forms) through social networks (Facebook, emails) and direct visits to dentists at their offices.

### Sample size calculation

According to data from the Mexican government, the population of dentists in the study area is 70,000 [31]. To calculate a sample with 50% heterogeneity, 5% margin of error, and 95% confidence level, we used the Netquest online calculator (https://www.netquest.com/en/sample-size-calculator), which gave us a sample size of 387. Acceptance of responses to the questionnaire was stopped on January 4, 2023, once the required sample size was reached.

### Survey instrument and variables

We used the Spanish version of the questionnaire by Gambetta–Tessini et al. [23] to assess the knowledge, perceptions, and clinical experiences of dentists from the metropolitan area of Mexico City about MIH. Investigation of the perceptions and knowledge of MIH included clinical experience, treatment, views on aetiology, and the need for further training in MIH management. The questionnaire consisted of 30 questions divided into six sections: Sect. 1- socio-demographic and professional information; Sect. 2- knowledge of MIH; Sect. 3- clinical appearance and distribution of MIH; Sect. 4- aetiologic factors; Sect. 5: clinical management considerations; and Sect. 6: aspects of continuing education and improvement. After modifying the questionnaire from Chilean Spanish to Mexican Spanish, a pilot version of the questionnaire was circulated among 15 dentists who were subsequently not included in the final sample population.

### Data analysis

The information of each participant was captured in Excel spreadsheets, and the data was transferred to the SPSS version 21.0 package (IBM Corp., Armonk, NY, USA). The analysis provided comparisons between general practice dentists (GPDs), paediatric dentists (PDs), and dentists of other specialities (DoSs) based on the distribution of selected biographical, educational, and work experience variables. Descriptive statistics were determined, and the chi-squared test was used for nominal or ordinal variables. Results were considered significant at the < 0.05 alpha level.

## Results

Responses to the questionnaire were received from 391 dentists (38% response rate); 66.2% were women. A total of 184 (47%), 99 (25.3%), 54 (13.4%), 24 (6.1%), and 29 (7.4%) surveyed dentists were in the age ranges of 20–29, 30–39, 40–49, 50–59, and more than 60 years, respectively. Most of the respondents had a professional practice of –-9 years (n = 234). A total of 306 (78%) of the surveyed population worked in the private sector, 25 (6.4%) in the public sector, and 58 (15%) simultaneously in the private and public sectors. In terms of workplace characteristics, 363 (93%) worked in urban areas and 27 (7%) in rural areas. The number of surveyed GPDs, PDs, and DoSs was 224, 67, and 100, respectively (Table 1).

**Table 1:** Demographic characteristics of study participants

	Total	GDP (%)	PD (%)	DoS (%)
	N = 391	N = 224	N = 67	N = 100
	n (%)	n (%)	n (%)	n (%)
**Sex**				
Female	259 (66.2)	147 (65.6)	52 (77.6)	60 (60)
Male	132 (33.8)	77 (34.4)	15 (22.4)	40 (40)
**Age**				
≤ 29	184 (47)	159 (71)	14 (20.9)	11 (11)
30–39	99 (25.3)	28 (12.5)	29 (43.3)	42 (42)
40–49	54 (13.8)	23 (10.3)	7 (10.5)	24 (24)
50–59	24 (6.1)	7 (3.1)	8 (11.9)	9 (9)
> 60	29 (7.4)	7 (3.1)	8 (11.9)	14 (14)
**Years in practice**				
< 9	234 (59.8)	177 (79)	27 (40.3)	30 (30)
10–19	77 (19.7)	26 (11.6)	19 (28.4)	32 (32)
20–29	33 (8.4)	9 (4)	6 (9)	18 (18)
30–39	26 (6.6)	6 (2.7)	11 (16.4)	9 (9)
< 40	21 (5.4)	6 (2.7)	4 (6)	11 (11)
**Type of practice**				
Private	306 (78.3)	197 (87.9)	43 (64.2)	66 (66)
Government	25 (6.4)	11 (4.9)	4 (6)	10 (10)
Both	58 (14.8)	14 (6.3)	20 (30)	24 (24)
**Location of main practice**				
Urban	363 (92.8)	207 (92.4)	63 (94)	93 (93)
Rural	27 (6.9)	17 (7.6)	4 (6)	6 (6)

General Dental Practitioner, Paediatric Dentist, Dentists of other Specialties

### MIH identification

A total of 97% of the PDs identified the images as those of MIH, while only 85% and 83% of the GDP and other specialist populations, respectively, could identify the disease (*p* = 0.177). In terms of experience with MIH patients, 99% of PDs had treated patients with MIH, unlike only 75% of GDPs (*p* < 0.001). The MIH lesions were observed as white demarcated opacities in 48–52% of cases by GDPs and DoSs, while 57% of lesions were detected as yellow/brown demarcated opacities by PDs. Approximately 20% of the participating PDs detected the lesions in post-eruptive enamel breakdown. A total of 90%, 30%, and 33% of PDs, GPDs, and DoSs, respectively, believed that the incidence of MIH has increased (< 0.001) (Table 2).

**Table 2:** MIH perception, clinical appearance, and prevalence according to study participants

Question	Total N = 391	General Practice Dentists N = 224	Paediatric Dentists N = 67	Dentists of other Specialties N = 100	*p*-value
	N (%)	N (%)	N (%)	N (%)	
**Image recognition**					0.177
MIH	338 (86%)	190 (85%)	65 (97%)	83 (83%)	
Enamel hypoplasia	39 (10%)	24 (11%)	2 (3%)	13 (13%)	
Fluorosis	12 (3%)	9 (4%)	0	3 (3%)	
Amelogenesis	2 (0.5%)	1 (4%)	0	1 (1%)	
**Do you notice hypomineralised teeth in your practice?**					
Yes	327 (84%)	169 (75%)	66 (99%)	92 (92%)	< 0.001*
**What do you most frequently notice in your practice?**					< 0.001*
White demarcated opacities	179 (46%)	116 (52%)	15 (22%)	48 (48%)	
Yellow/brown demarcated opacities	185 (47%)	99 (44%)	38 (57%)	48 (48%)	
Post-eruptive enamel breakdown	27 (7%)	9 (4%)	14 (21%)	4 (4%)	
**Do you perceive that the incidence of MIH has increased in recent years?**					< 0.001*
Yes	159 (41%)	66 (30%)	60 (90%)	33 (33%)	
No	143 (37%)	95 (42%)	4 (6%)	44 (44%)	
Don’t know	89 (23%)	63 (28%)	3 (5%)	23 (23%)	
**How confident do you feel when diagnosing teeth with MIH?**					< 0.001*
Very confident	88 (23%)	28 (13%)	38 (57%)	22 (22%)	
Confident	210 (54%)	129 (58%)	24 (36%)	57 (57%)	
Unconfident	90 (23%)	66 (30%)	4 (6%)	20 (20%)	
Very unconfident	3 (1%)	1 (4%)	1 (2%)	1 (1%)	
**Do you think that a significant percentage of caries is due to the presence of MIH?**					0.006*
Yes	239 (61%)	131 (59%)	39 (58%)	69 (69%)	
No	100 (26%)	53 (24%)	25 (37%)	22 (22%)	
Don’t know	52 (13%)	40 (18%)	3 (5%)	9 (9%)	
**Do you think the pattern of caries related to MIH is different from the classical caries pattern?**					0.014*
Yes	298 (76%)	160 (71%)	58 (87%)	80 (80%)	
No	50 (13%)	35 (16%)	8 (12%)	7 (7%)	
Don’t know	43 (11%)	29 (13%)	1 (2%)	13 (13%)	
**Have you been aware of the fact that MIH is a developmental defect that differs from fluorosis and hypoplasia?**					
Yes	361 (92%)	202 (90%)	67 (100%)	92 (92%)	0.03*
**How prevalent do you think MIH might be in your community? (One option chosen)**					0.001*
0–10%	157 (40%)	91 (41%)	12 (18%)	54 (54%)	
11–20%	65 (17%)	33 (15%)	15 (22%)	17 (17%)	
21–30%	70 (18%)	42 (19%)	15 (22%)	13 (13%)	
31–40%	42 (11%)	23 (10%)	11 (16)	8 (8%)	
< 41%	55 (14%)	34 (15%)	13 (20%)	8 (8%)	
**Do you think it would be worthwhile investigating the prevalence?**					
Yes	384 (98%)	219 (98%)	67 (100%)	98 (98%)	0.474
**Do you think MIH is a clinical problem?**					
Yes	384 (98%)	219 (98%)	67 (100%)	98 (98%)	0.474
**What is the severity of this problem according to you in your community?**					0.001*
Mild	98 (25%)	62 (28%)	5 (8%)	31 (31%)	
Moderate	190 (49%)	107 (48%)	42 (63%)	41 (41%)	
Severe	61 (16%)	28 (13%)	17 (25%)	16 (16%)	
Not sure	42 (11%)	27 (12%)	3 (5%)	12 (12%)	

General Dental Practitioner, Paediatric Dentist, Dentists **of other Specialties.** *Statistically significant (*p* < 0.05), Pearson’s chi-squared (χ^2^) test

### Perception regarding MIH aetiology

In total, 275 (70%) of the respondents thought that genetic factors participate in the aetiology of MIH, and 254 (65%) believed that the antibiotics or medications administered to the mother or child are aetiological factors of MIH. The least associated factor was fluoride (n = 72). Most of the participants believed that insult occurs during pregnancy (n = 278, 71%). In addition, 55%, 36%, and 38% of PDs, GPDs, and DoSs, respectively, believed that insults causing MIH may occur during the first year of life (*p* < 0.001) (Table 3).

**Table 3:** Knowledge or perception about the aetiology of MIH

Question	Total N = 391	General Practice Dentists N = 224	Paediatric Dentists N = 67	Dentists of othe Specialties N = 100	*p*-value
	N (%)	N (%)	N (%)	N (%)	
**Which factors do you think are involved in the aetiology of MIH? (you can select more than one option)**					
Genetic factors	275 (70)	169 (75)	40 (62)	66 (66)	0.047*
Antibiotics or medications	254 (65)	144 (64)	43 (64)	67 (67)	0.088
Chronic medical conditions affecting the mother or child	237 (61)	140 (63)	47 (70)	50 (50)	0.022*
Acute medical conditions affecting the mother or child	192 (49)	109 (49)	39 (58)	44 (44)	0.019*
Environmental contaminants	143 (37)	75 (34)	32 (48)	36 (36)	0.103
Fluoride exposure	72 (19)	47 (21)	4 (6)	21 (21)	0.015*
**During what time/period do you think this insult occurs? (you can select more than one option**					
During pregnancy	278 (71)	159 (71)	54 (81)	65 (66)	0,112
During first year of life	139 (36)	64 (29)	37 (55)	38 (38)	< 0.001*
During second year of life	47 (12)	24 (11)	9 (13)	14 (14)	0,651
Not sure	57 (15)	37 (17)	3 (5)	17 (17)	0,036

General Dental Practitioner, Paediatric Dentist, **Dentists of other Specialties.** *Statistically significant (*p* < 0.05), Pearson’s chi-squared (χ^2^) test

### Considerations for clinical management of MIH

A total of 61% (n = 240) dentists, especially PDs (88%, *p* < 0.001), believed that parents play an important role in the management of MIH. Similarly, 88% of PDs were found to be comfortable treating children with MIH (*p* > 0.001). Glass ionomer cement, resin composites, and resin-modified glass ionomers were the most widely used biomaterials by all groups of dentists. PDs were found to be using infiltrating resins more often compared to other dentist groups (*p* < 0.05). In terms of the problems associated with the management of MIH, 86% of respondents believed limited training to be the main problem. A total of 8 (12%) PDs were reported to face difficulty in achieving good local anaesthesia, while the other groups of dentists ranged from 2 to 3% (*p* < 0.001) (Table 4).

**Table 4:** Considerations for the clinical management of MIH

Question	Total N = 391	General Practice Dentists N = 224	Paediatric Dentists N = 67	Dentists of other Specialties N = 100	*p*-value
	N (%)	N (%)	N (%)	N (%)	
**Do you think parents can play a role in the management of MIH?**	240 (61)	121 (54)	59 (88)	60 (69)	< 0.001
**Do you feel comfortable providing treatment for a child with MIH?**					
Yes	240 (61)	121 (54)	59 (88)	60 (69)	< 0.001
**Do you consult the paediatric dentist for children with any sign of MIH?**					
Yes	230 (59)	155 (69.2)		75 (75)	0.288
**Do you think early detection is important to treat MIH?**					
Yes	381 (97)	217 (97)	67 (100)	97 (97)	0.695
No	4 (1)	3 (1)	0	1 (1)	
Not sure	6 (2)	4 (2)		2 (2)	
**What type of biomaterial do you use most frequently to treat these teeth? (you can select more than one option)**					
GIC	184 (47)	99 (44)	33 (49)	52 (52)	0.397
Composite resin	134 (34)	71 (32)	25 (37)	38 (38)	0.46
Resin-modified glass ionomer	179 (46)	106 (48)	32 (48)	41 (41)	0.568
Compomer	43 (11)	19 (9)	9 (13)	15 (15)	0.18
Amalgam	19 (5)	11 (5)	3 (5)	5 (5)	0.987
Stainless steel crown	148 (38)	80 (36)	28 (42)	40 (40)	0.605
Resin infiltrant	81 (21)	39 (18)	21 (31)	21 (21)	0.049*
**Which of the following options may be a problem for managing MIH teeth? (you can select more than one option).**					
Dental treatment that needs a long time to be accomplished	144 (37)	83 (37)	24 (36)	37 (38)	0.96
Child’s behaviour	168 (43)	99 (44)	26 (39)	43 (43)	0.72
Difficulty in achieving local anaesthesia	16 (4)	5 (2)	8(12)	3 (3)	0.001
Insufficient training to treat children with MIH	337 (86)	188 (84)	63 (94)	86 (86)	0.124

General Dental Practitioner, Paediatric Dentist, **Dentists of other Specialties.** *Statistically significant (*p* < 0.05), Pearson’s chi-squared (χ^2^) test

### Aspects of continued education and improvement

A total of 57% of surveyed dentists had received information regarding MIH; among the PDs, 97% had knowledge of MIH (*p* < 0.002). The most widely used source of information was the Internet, and notably, PDs expressed a greater need for additional information on treatment topics (*p* = 0.049) (Table 5).

**Table 5:** Aspects of continuing education and improvement

Question	Total N = 391	General Practice Dentists N = 224	Paediatric Dentists N = 67	Dentists of other Specialties N = 100	*p*-value
	N (%)	N (%)	N (%)	N (%)	
**Have you received any information on molar incisor hypomineralisation?**					
Yes	222 (57%)	107 (51%)	64 (97%)	51 (51%)	< 0.002*
**Which is/are your source(s) of information? (you can select more than one option).**					
Internet	140 (36%)	78 (37%)	26 (40%)	36 (36%)	0.871
Dental Journals	110 (28%)	48 (23%)	23 (35%)	39 (39%)	0.008*
Books	81 (21%)	44 (21%)	12 (18%)	25 (25%)	0.551
Continuing education courses	119 (30%)	54 (26%)	25 (39%)	40 (40%)	0.072
**Where do you think more information is necessary? (you can select more than one option)**					
Aetiology	234 (60%)	138 (66%)	41 (62%)	55 (55%)	0.173
Diagnosis	280 (72%)	166 (79%)	45 (68%)	69 (69%)	0.072
Treatment	328 (84%)	185 (88%)	62 (94%)	81 (81%)	0.049*

General Dental Practitioner, Paediatric Dentist, **Dentists of other Specialties.** *Statistically significant (*p* < 0.05), Pearson’s chi-squared (χ^2^) test

## Discussion

This is the first study that addresses the knowledge, clinical experience, and perceptions of MIH in a population of dentists in Mexico. A total of 47% of the surveyed dentists were in the age range of 20–29 years; greater participation from this age group can be attributed to the familiarity of participants with digital platforms such as Facebook groups and Google Forms, which may have sped up the response to a large extent and motivated the dentists to complete the survey [32]. The majority of the participants were women (66%), which may have been due to the feminisation of the healthcare professions that have been reported in different countries [33, 34].

### MIH identification

The surveyed dentists, mostly PDs (99%), had experience of MIH lesions. These data are similar to those reported in other studies [19, 21, 23, 29]. A total of 85% of the respondents identified the lesions on the survey images as MIH lesions, 10% as enamel hypoplasia, and only 1% identified them as fluorosis. The foregoing study suggests that, based on their clinical experience, the majority of respondents recognized the delimited lesions of dental enamel hypomineralisation, a characteristic feature of MIH, in a manner consistent with the data from the survey administered to students at the Medical University of Vienna last year [35]. In the present study, lesions with yellow-brown opacity were found to be more prevalent and PDs had observed post-eruptive fractures in the enamel as manifestations of MIH. In a study conducted in Norway, all respondents reported having encountered MIH in their clinical practice, and yellow/brown demarcated opacities were encountered slightly more frequently than white demarcated defects. Post-eruptive enamel breakdown was also observed. This data is similar to that of our study [36]. Other studies conducted in different countries also report greater prevalence of the yellow-brown lesion [19, 21, 23, 28–30].

In this study, most of the PDs perceived an increased incidence of MIH, while only 25% of the other responding dentists reported similarity in data to that of Iraq [19]. The vast majority of the Australian GDPs (84.6%) and Oral Healthcare Practitioners (81.6%) reported that they perceived an increased incidence of MIH during their professional lifetime, in Chile, 52.4% of GPDs reported the same [23].

The published epidemiological studies indicate that the prevalence of MIH in Mexico City and its metropolitan area ranged from 14 to 42%, with an average of 29.6%. Therefore, the responses of 39% of GPDs, 42% of PDs, and 66% of other specialists are within the reported prevalence range [6–12].

In our study, the confidence level for diagnosing MIH lesions was very high among PDs, while it was high among GPDs and other specialists. These data are similar to those reported in the UK in 2016 [22], which stated that most respondents feel very confident or confident in the diagnosis of this condition. Similarly, Iranian dentists were found to be confident in correctly diagnosing MIH lesions. Approximately 87% of PDs believed that the pattern of MIH-related caries is different from the classic one.

### Perception regarding MIH aetiology

The aetiology of MIH remains unclear and may have a multifactorial aetiology. The results of different meta-analyses are not conclusive for the different factors associated with the aetiology of MIH [37]. However, recent studies have shown that there is a genetic background that is modulated by epigenetic factors associated with MIH and that perinatal and postnatal etiological factors increase the chances of developing MIH more than prenatal factors [38].

In our study, the respondents considered that genetic factors are responsible for MIH, they also associated antibiotics administered to the mother or child and chronic and acute medical conditions of the mothers (in pregnancy) or children with MIH. Some respondents also considered exposure to environmental pollutants and fluoride to be an etiological factor.

Similarly, in other studies carried out in different countries [18, 19, 26–28], the dentists’ responses showed a tendency to consider the aetiology of MIH as multifactorial. The association of MIH with the consumption of medications by the mother during pregnancy or the newborn, as well as acute or chronic medical conditions of the mother or newborn during the period of formation of teeth affected by MIH, reinforces the idea of multifactorial pathogenesis in MIH.

In this study, many participants stated that they believe that the development of MIH is due to genetic pathogenesis. Therefore, they would agree with the findings of the study conducted between monozygotic and heterozygotic twins, in which there was greater concordance in the diagnosis of MIH between monozygotic twins [13]. It is important to note that environmental contaminants have been identified as aetiological factors in some studies through a relationship between environmental toxins and enamel development defects [39].

A total of 71% of the surveyed dentists considered that the alteration that leads to MIH occurs during pregnancy. However, in the study conducted in Iraq [19], only 42% of participants reported that the alteration occurs during the gestation period. In our work, 36% of the dentists considered that the alteration occurs between the first and second year of the child’s life, which was similar to the findings of the study conducted among PDs in the North American Midwest [24]. According to this study, 35% of dentists chose the first year of life as the period in which the aggression occurs.

### Considerations for clinical management of MIH

A total of 97% of the respondents affirmed that early diagnosis of MIH is important for treatment, especially for the management of affected molars, since rapid post-eruptive enamel breakdown can occur, leading to loss of enamel and acute symptoms, thus complicating treatment [40]. Overall, 88% of PDs considered that parents play an important role in the management of MIH, since by taking children to the dentist on time preventively or quickly before the first symptoms appear, the dentist can make an early diagnosis. Parents also make an important contribution by participating in following dental health care instructions. Of note, most PDs were found to be comfortable treating children with MIH. Similarly, in the study conducted among Egyptian dentists, PDs were comfortable treating children with MIH [41]. In Oslo, 68% of dentists were confident while treating children with MIH; this finding is similar to the data collected from all dentists in our survey, wherein 61% reported feeling comfortable caring for children with MIH [36]. In our survey, 59% of those surveyed mentioned consulting a PD as soon as they found a child with signs of MIH; this number is similar to that reported in the study conducted in Malaysia and Egypt [20, 41]. In Norway, less than a third of the respondents (27.8%) had referred patients with teeth affected by MIH to specialists in paediatric dentistry [42].

The best practice guideline for clinicians treating children with MIH indicates that the therapeutic approach will depend on the severity of the defect and the patient’s age. Thus, for mild cases, the use of sealants and casein phosphopeptide-amorphous calcium fluoride phosphate (CPP-ACFP) is recommended, but as the cases worsen and the molars make their eruption in the oral environment, glass ionomers are recommended, and once the molars are erupted and functional, composite resins are recommended. If the molars present extensive lesions caused by post-eruptive enamel breakdown and the development of extensive caries, it is recommended to use preformed metal crowns; however, when the child is between 8 and 10 years of age and coronary destruction has occurred, the aforementioned materials cannot be used, so the affected molar extracted [17].

The most widely used material by the surveyed dentists for the treatment of teeth affected by MIH is glass ionomer (n = 184), followed by resin-modified glass ionomer (n = 179) and composite resins (n = 134). Similarly, the results of the study conducted among PDs in Australia and New Zealand report that the most used materials were glass ionomers and composite resins [18]. Among Egyptian dentists [41], composite resin was the most popular (74%), followed by resin-modified glass ionomer (48.2%) and preformed crowns (41.6%).

When it comes to the challenges encountered in managing MIH cases, 168 dentists (43%) identified uncooperative child behaviour, 337 (86%) expressed concerns about the limited training available for MIH, and 16 (4%) highlighted the extended duration of treatment as the primary issues. Only a small percentage of participants believed that difficulty in achieving adequate local anaesthesia was a contributing factor. Dentists from Kuwait [24] also identified child behaviour as the most common barrier to the management of MIH; therefore, they reflected the inadequate training of GPDs in child management, while the North American PDs selected the long duration of restorative treatments as the most common clinical problem [26]. The Greek dentists stated that children with MIH-affected teeth were 2 to 5.5 times more likely to report difficulty in achieving sufficient anaesthesia and face hypersensitivity problems [30].

Access to information regarding MIH is facilitated by the type of training. 97% of the PDs stated that they had received information about MIH, in contrast to only 51% of other specialists and GPDs. In Norway, 87% of dentists and dental hygienists reported to have received information about MIH [36].

### Aspects of continuing education and improvement

In the survey, 140 dentists (36%) reported using the Internet, 119 (33%) preferred courses, and 110 (28%) relied on dental magazines or journals as their sources of information on MIH. Remarkably, all participants expressed a strong interest in obtaining more knowledge about MIH. In Saudi Arabia, the responding dentists mentioned that 40% of dentists with less than 5 years of experience obtained information from Saudi journals [21]. In the case of dentists from Kuwait, the main source of information for general dentists (37%) was found to be the Internet, while PDs (63%) used magazines or journals [24].

A total of 86% of the dentists stated that they would like to receive a clinical update course in relation to MIH. Respondents to this survey and a greater proportion of PDs selected the treatment topic more frequently (83%). Additionally, in Malaysia, general dentists indicated the need for more clinical training for the management of MIH in relation to diagnosis and treatment modalities [20].

It is essential to acknowledge certain limitations of this study. We observed substantial participation from general practitioners and other specialists, but a greater involvement of PDs would have been desirable. In addition, the study was limited to an area of Mexico, limiting the generalisability of its findings. The results reveal that there is awareness to a certain extent about this type of enamel alteration; however, there is a need for more training, especially in terms of the treatment. Therefore, the results should be incorporated into the courses on the topics related to MIH. The higher education institutions in Mexico should be alerted to generate courses that offer training on MIH and address this need for continuing education, thereby helping dentists = deal with MIH in a better way.

## Conclusions

Approximately 80% of the dentists mentioned having observed MIH lesions in their practice. They considered that the aetiology of MIH was multifactorial, with a strong genetic component and the disrupting agent acting during pregnancy. Most considered MIH to be of low prevalence. Compared with general dentists, PDs perceived a higher percentage of MIH occurrences to have increased in their practice. The material most used by the dentists surveyed was glass ionomer. Most dentists indicated that the main problems for the management of MIH are uncooperative children’s behaviour and lack of training.

All clinicians need to be educated and trained in the correct forms to detect and diagnose this defect in early stages. This will help to achieve a correct and timely diagnosis and establish a personalised and adequate treatment for each patient. However, on the basis of the results, it is necessary to develop updated courses that address the issue of aetiology, diagnosis, and management of MIH lesions based on international guidelines for the clinical management of patients with MIH.

## Data Availability

The datasets used and/or analysed during the current study are available from the corresponding author on reasonable request.

## List of Abbreviations

DoS: Dentist of other Specialties
GPD: General Practice Dentists
MIH: Molar-incisor hypomineralisation
PD: Paediatric Dentists
